# Plant-Derived Saponin and Polysaccharide Vaccine Adjuvants: Structure–Activity Relationships and Immune-Enhancement Strategies

**DOI:** 10.3390/vaccines14100890

**Published:** 2026-10-11

**Authors:** Yuxin Lao, Zhengxiang Wang, Ying Tian, Jianhua Sun, Likun Gong

**Affiliations:** 1 School of Pharmacy, Guangdong Pharmaceutical University, Guangzhou 510006, China; laoyuxin1723@zidd.ac.cn; 2 Zhongshan Institute for Drug Discovery, Shanghai Institute of Materia Medica, Chinese Academy of Sciences, Zhongshan 528400, China; wangzhengxiang8534@njucm.edu.cn (Z.W.); tianying0585@zidd.ac.cn (Y.T.); 3 School of Chinese Materia Medica, Nanjing University of Chinese Medicine, Nanjing 210023, China; 4 State Key Laboratory of Drug Research, Shanghai Institute of Materia Medica, Chinese Academy of Sciences, Shanghai 201203, China; 5 Innovation Center for AI and Drug Discovery, School of Pharmacy, East China Normal University, Shanghai 200062, China

**Keywords:** plant-derived adjuvants, structure–activity relationships, immune-enhancement strategies, saponins, polysaccharides, delivery systems, vaccine adjuvants

## Abstract

Plant-derived compounds, particularly saponins and polysaccharides, have attracted interest as vaccine adjuvants because of their diverse immunomodulatory activities. Increasing evidence suggests that the immunological activities of these compounds are closely associated with their structural characteristics, highlighting the importance of understanding how structural features influence their interactions with the immune system. Nevertheless, a systematic understanding of the relationships between structural features and immunological activity remains incomplete, limiting the rational development and optimization of plant-derived adjuvants. In addition, their broader development and translation are constrained by challenges such as poor stability, low bioavailability, insufficient immunostimulatory activity, and limited delivery efficiency. Against this background, this review focuses on the structure–activity relationships of plant-derived saponin and polysaccharide adjuvants, with particular emphasis on how key structural features influence the engagement of pattern recognition receptors (PRRs), antigen uptake and presentation, cytokine production, and subsequent humoral and cellular immune responses. Building on these structure–activity relationships, we further discuss current strategies for improving adjuvant performance, including structural modification and derivatization, delivery-system optimization, and combination strategies. Together, these insights provide a structure–activity framework for the rational design and development of more effective next-generation plant-derived saponin and polysaccharide adjuvants.

## 1. Introduction

Vaccination is one of the most effective public health interventions for preventing infectious diseases, reducing the burden of disease, and establishing herd immunity. In recent years, advances in antigen structural biology, nucleic acid technology, and materials science have driven a gradual shift in vaccine development from the use of whole pathogens to platforms with well-defined, engineerable components, such as recombinant proteins, epitope peptides, virus-like particles, and nucleic acid vaccines [1]. Compared with attenuated or inactivated vaccines, subunit vaccines generally offer improved safety profiles, greater manufacturing control, and greater flexibility in antigen design. However, highly purified antigens often lack the innate immune stimulatory signals carried by the pathogen itself, and they may fail to elicit sufficiently potent and durable protective immunity when administered alone [2]. Adjuvants play an important role in compensating for the limited immunogenicity of such vaccines and in inducing and maintaining effective protective immunity [3]. Therefore, how to select an adjuvant with both efficacy and safety according to different antigenic characteristics and immune protection needs has become a key issue in modern vaccine development.

At present, adjuvants that have been used in human vaccines or in clinical development mainly include aluminum-based adjuvants, squalene-based oil-in-water emulsions such as MF59 and AS03, pattern recognition receptor (PRR) agonists such as 3-O-desacyl-4′-monophosphoryl lipid A (MPL) and CpG 1018, saponin-based particulate adjuvants such as Matrix-M, and combination adjuvant systems such as AS01 and AS04 [4,5]. Liposomes, polymers and inorganic particles are mainly used as delivery platforms for new adjuvants [6]. Despite substantial progress in adjuvant development, several important challenges remain to be addressed. For example, the induction of robust CD8+ T cell, mucosal, and tissue-resident memory responses remains challenging [7]. Immunosenescence in older adults and impaired immune function in immunocompromised individuals may reduce responsiveness to adjuvant stimulation [8,9,10]. Therefore, there is a pressing need to develop more advanced and versatile vaccine adjuvants.

In recent years, accumulating evidence indicates that plant-derived natural products with diverse chemical structures possess substantial immunostimulatory potential, providing a diverse molecular resource for the discovery and optimization of new vaccine adjuvants, among which saponins and polysaccharides are the two most extensively studied candidates [11,12]. Incorporation of QS-21 into several licensed vaccine adjuvant systems demonstrates that plant-derived saponins can be successfully translated into clinical use. Mechanistic studies indicate that QS-21 can promote the activation of antigen-presenting cells (APCs), intracellular membrane perturbation, antigen cross-presentation, and antigen-specific T-cell responses, although these effects depend strongly on formulation and co-adjuvant context [13]. Plant polysaccharides usually have complex immunomodulatory pathways. They can activate innate immune responses, particularly through PRR-associated pathways in APCs, including signaling associated with Toll-like receptor 2 (TLR2), Toll-like receptor 4 (TLR4), and Dectin-1 [11,14]. In addition, some plant-derived adjuvants can promote the formation of memory T cells, thereby helping to establish and maintain immune memory [11]. Plant-derived adjuvants can also improve antigen presentation and immune response in the context of immune senescence [11], and can promote mucosal secretory immunoglobulin A (sIgA) and local antigen-specific T and B cell responses, thus showing their potential application as mucosal vaccine adjuvants [14,15]. These multi-level immune effects show the research prospect of plant saponins and polysaccharides as vaccine adjuvants. More importantly, these effects are not determined by their plant origin alone, but are constrained by their chemical structure. Structural features of saponins, such as the aglycone core, sugar chains, and acyl side chains, and of polysaccharides, such as molecular weight, monosaccharide composition, glycosidic linkages, branching patterns, and conformations, may influence receptor recognition, APC activation, cytokine secretion, and downstream adaptive immune responses. Therefore, it is necessary to review the existing research on plant-derived adjuvants from the perspective of structure–activity relationships, identify the key structural factors that influence their immune function, and provide a basis for subsequent structural modification, delivery system optimization, and combination-adjuvant design.

Based on the above understanding, this review discusses the structure–activity relationships and immune-enhancement strategies of plant-derived saponins and polysaccharides. Firstly, we analyze how key structural factors affect the immune activity and establish the structure–activity relationship framework of saponins and polysaccharides. Secondly, we summarize the immune-enhancement strategies around structural modification and derivatization, optimization of delivery systems, and combination adjuvants, and discuss how these strategies can improve stability, bioavailability, immune bias, and safety while enhancing immune efficacy. Finally, we propose future research directions from the perspective of structure–activity relationship and standardized evaluation, aiming to provide valuable insights for the development of more precise, reproducible and translatable plant-derived saponin and polysaccharide adjuvants.

## 2. Plant-Derived Saponins

Plant-derived saponins represent an important class of immunostimulatory vaccine adjuvant candidates. Structurally, they generally consist of a hydrophobic triterpenoid or steroidal aglycone linked to one or more hydrophilic sugar chains. Some saponins additionally contain a fatty acyl side chain. Together, the aglycone core, sugar chains, and fatty acyl side chain determine adjuvant activity while also influencing hemolytic toxicity. Therefore, the structure–activity relationship of saponins cannot be attributed to a single functional group or a simple hydrophilic-lipophilic balance [13,16,17].

The saponins of *Quillaja saponaria* Molina are the most typically used adjuvant components among plant-derived saponins. Highly purified QS-21, together with MPL, constitutes the AS01 adjuvant system, which has been used in Shingrix (AS01B), RTS,S/AS01E and Arexvy (AS01E) [18]. Matrix-M, used in NVX-CoV2373 and R21/Matrix-M, comprises two distinct nanoparticle populations containing different purified *Quillaja* saponin fractions formulated with cholesterol and phospholipids [18,19]. Natural QS-21 is mainly composed of QS-21-Api and QS-21-Xyl, which differ only in the terminal sugar residue of the C28 oligosaccharide. Its structure can be divided into four interdependent regions: aglycone core, C3 branched trisaccharide, C28 linear tetrasaccharide, and glycosylated acyl side chain [17].

QS-21 does not appear to act through a single identified cell-surface PRR. Instead, its adjuvant activity involves formulation-dependent intracellular stress and coordinated interactions among immune cells in the draining lymph nodes. Using QS-21 incorporated into cholesterol-containing liposomes, Detienne et al. found that QS-21 rapidly reached the draining lymph nodes and accumulated predominantly in CD169+ sinus macrophages. These macrophages initiated innate immune cell recruitment and the maturation of dendritic cells (DCs) through the integration of caspase-1- and MyD88-dependent signaling, partly mediated by local high-mobility group protein B1 (HMGB1) release, whereas Batf3-dependent DCs were required for subsequent antigen-specific T cell priming [20]. Using QS-21 formulated in 1,2-dioleoyl-sn-glycero-3-phosphocholine (DOPC)/cholesterol liposomes, Welsby et al. further showed that, in human monocyte-derived DCs, cellular uptake of QS-21 required membrane cholesterol and was followed by its accumulation in lysosomes and lysosomal destabilization, leading to inflammatory activation dependent on cathepsin B and spleen tyrosine kinase (Syk). Cathepsin B also contributed to QS-21-enhanced antigen-specific CD4+ and CD8+ T-cell responses in vivo [21]. More recently, Lv et al. showed that unformulated QS-21 could promote phagolysosomal membrane perturbation, cytosolic antigen translocation, major histocompatibility complex class I (MHC-I) cross-presentation, and antigen-specific CD8+ T-cell activation, further connecting intracellular membrane stress with cellular immunity [22].

Additional mechanisms arise from the cooperation between QS-21 and MPL within the AS01 adjuvant system. Marty-Roix et al. showed that unformulated QS-21, following monophosphoryl lipid A (MPLA)-mediated priming, activated the ASC–NLRP3–caspase-1 inflammasome in murine macrophages and DCs, resulting in IL-1β and IL-18 release. By contrast, in vivo, NLRP3-deficient mice immunized with QS-21-adjuvanted HIV-1 gp120 developed stronger antigen-specific Th1- and Th2-associated T-cell responses and higher IgG1 and IgG2c levels than wild-type mice [23]. In the complete liposomal AS01 system, Coccia et al. demonstrated that QS-21 and MPL generated synergistic innate responses that were not reproduced by either component alone. QS-21-associated cellular stress and IL-18 signaling cooperated with MPL-mediated TLR4 activation and IL-12 production, inducing an early burst of IFN-γ predominantly from NK cells and innate-like CD8+ T cells. This early response subsequently promoted polyfunctional, Th1-biased antigen-specific CD4+ T-cell responses and Th1-associated antibody isotype switching [24]. Using intact human lymph node explants, Stylianou et al. recently confirmed that AS01-induced cytokine production and DC maturation depended on the spatially and temporally coordinated activation of sinus macrophages, monocytes, and DCs, supporting the multicellular nature of this mechanism in human lymphoid tissue [25]. Collectively, QS-21 itself primarily contributes to lymph node targeting, lysosomal stress, inflammatory signaling, and antigen cross-presentation, whereas its combination with MPL in AS01 establishes an additional IL-12/IL-18–IFN-γ axis that amplifies and directs Th1-biased humoral and cellular immunity.

**Figure 1:**
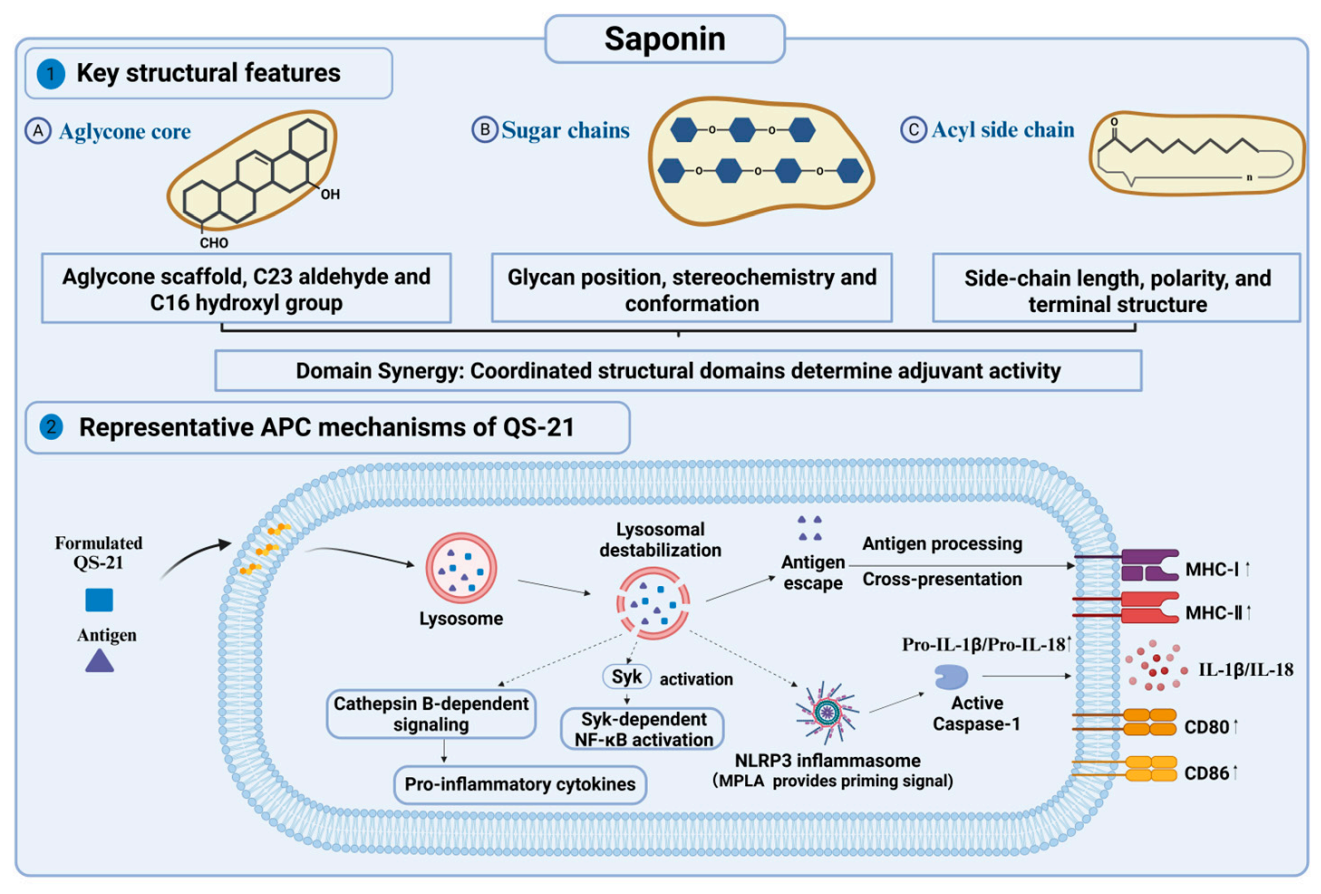
Structure–activity relationships of plant-derived saponins and representative APC mechanisms of QS-21. Solid arrows indicate experimentally supported effects or functional dependencies, whereas dashed arrows indicate proposed mechanistic links that remain incompletely resolved. The symbol ↑ denotes increased expression or levels of the indicated molecules. APC, antigen-presenting cell; CD80, cluster of differentiation 80; CD86, cluster of differentiation 86; IL-1β, interleukin-1 beta; IL-18, interleukin-18; MHC-I, major histocompatibility complex class I; MHC-II, major histocompatibility complex class II; MPLA, monophosphoryl lipid A; NF-κB, nuclear factor kappa B; NLRP3, NOD-, LRR- and pyrin domain-containing protein 3; Pro-IL-1β, precursor of interleukin-1 beta; Pro-IL-18, precursor of interleukin-18; Syk, spleen tyrosine kinase.

The potent adjuvant activity of QS-21 cannot be attributed to any single structural feature, but more likely depends on the coordinated contributions of its constituent domains [17]. Comparison of natural saponins, semisynthetic derivatives, and structurally simplified analogues revealed that some structures traditionally seen as essential can be replaced or deleted, but their replaceability depends on the maintenance of appropriate spatial relationships among the remaining domains. Therefore, this section focuses on the most studied QS-21, supplemented by other plant-derived saponins, and discusses the structure–activity relationships of saponins according to the four levels of aglycone core, sugar chain, side chain, and domain synergy (Figure 1).

### 2.1. Triterpene Aglycone Core: Shift from “Essential Aldehyde Groups” to Structural Background Dependence

The structure in the triterpene aglycone core of QS-21 that received earlier attention was the C23 aldehyde group attached to the C4 position of the aglycone core of quillaic acid. Early structure–activity studies found that the reduction or derivatization of this aldehyde group could be accompanied by reduced antibody responses, lymphocyte proliferation or antigen-specific cytotoxic T lymphocyte (CTL)-related effects, leading to the hypothesis that the C23 aldehyde might form Schiff-base adducts with amino groups on the T-cell surface and contribute to costimulation [13,16,17]. These results provide only indirect support for this model, and stable direct binding between QS-21 and CD2 or other receptors has not yet been demonstrated. Subsequent simplification of QS-21 analogues also weakens the interpretation that the C23 aldehyde group is absolutely essential. In specific structural scaffolds, this aldehyde group can be replaced by a hydroxymethyl, methyl, or oxime while retaining antibody-enhancing adjuvant activity [26,27]. Siddique et al. found that replacing the C23 aldehyde of a QS-21 analogue bearing a left-wing disaccharide and a C14 acyl chain with an oxime (VA-207) or a primary alcohol (VA-208) markedly reduced in vitro hemolytic activity relative to QS-21 across the tested concentrations. At the same 20 μg dose, neither analogue caused sustained body-weight loss during the seven-day post-injection observation period, while maintaining or enhancing ovalbumin (OVA)-specific antibody and splenic immune-cell responses [28]. In contrast, the reduction of the C23 aldehyde group to the primary alcohol abrogated OVA-specific IgG1 and IgG2a enhancement in the *Momordica* saponin-derived VSA-1 and VSA-2 [29]. This distinction suggests that the aldehyde contribution depends on the combination of aglycone scaffold, sugar, and side chains and cannot be directly extrapolated across saponin skeletons. Thus, the C23 aldehyde group is more suitable to be viewed as a structural variable affecting conformation and local reactivity rather than as an already identified receptor-binding group.

The aglycone scaffold appears to be a key determinant of the balance between adjuvant activity and toxicity of QS-21. Replacement of echinocystic acid with betulinic acid lacking the C16 hydroxyl group reduces hemolysis, but at the same time weakens IgG1 and CD8+ T-cell-related responses [30]. Reducing membrane damage does not necessarily preserve immune stimulation. Current research supports the view of the triterpene aglycone core as a scaffold that regulates three-dimensional conformation and membrane interactions rather than a collection of independent “active groups” [31].

### 2.2. Sugar Chains: Position, Stereochemistry and Conformation Are More Critical than Sugar-Residue Number

The two glycan domains of QS-21 appear to make unequal contributions to its adjuvant activity, and their effects cannot be explained solely by the total number of sugar residues. Chea et al. found that removal of the terminal apiose residue generated a C28 linear trisaccharide analogue with antibody-enhancing adjuvant activity comparable to that of synthetic QS-21-Api. Further truncation reduced antibody responses to keyhole limpet hemocyanin (KLH), OVA, and mucin 1 (MUC1), with the disaccharide analogue exhibiting increased toxicity and the monosaccharide analogue showing the lowest overall potency. These findings indicate that the terminal apiose residue is dispensable in this simplified scaffold, whereas retention of at least a linear trisaccharide at C28 may be important for maintaining a favorable balance between efficacy and toxicity [32]. In contrast, Fernández-Tejada et al. found that the entire C3 branched trisaccharide could be removed from an appropriately optimized QS-21-derived scaffold without substantially compromising adjuvant activity. The trisaccharide-deficient analogue SQS-0-0-5-18 induced anti-KLH, anti-MUC1, and anti-OVA IgG responses comparable to those elicited by natural and synthetic QS-21, while causing less vaccination-associated weight loss. Further optimization produced the minimal echinocystic acid derivative SQS-1-8-5-18, which retained potent adjuvant activity with markedly reduced toxicity [26]. Collectively, these findings suggest that the two glycan domains make unequal and scaffold-dependent contributions to QS-21 activity. The C3 branched trisaccharide may be dispensable in an optimized scaffold, whereas excessive truncation of the C28 linear oligosaccharide may compromise efficacy.

The central glycosidic linkage can influence the conformational preferences and adjuvant activity of QS-21 analogues. Walkowicz et al. showed that changes in linkage length, anomeric configuration, or flexibility frequently attenuated or abolished activity. Molecular dynamics simulations further indicated that active β-linked variants adopted more restricted torsional distributions around the central linkage, whereas less active analogues sampled broader or distinct conformational ensembles. Some hydrolytically stable linkage replacements retained activity, but chemical stability alone did not ensure adjuvant potency [33]. QS-7 analogues have also shown that local acetylation and internal linkage configuration of C28 glycans can alter IgG subclass distribution [34,35]. Collectively, these findings indicate that saponin glycans contribute to adjuvant activity by controlling molecular conformation and domain organization rather than merely increasing aqueous solubility.

The consequences of monosaccharide substitution are similarly context-dependent. Fuentes et al. found that replacing the terminal rhamnose–xylose moiety of a truncated QS-21-derived scaffold with β-linked lactose or cellobiose reduced anti-OVA IgG responses relative to the parent analogue. Molecular dynamics simulations associated this attenuation with greater conformational flexibility, although they did not establish a direct causal relationship [36]. In contrast, the naturally occurring QS-21-Rha variant, in which the terminal xylose of the C3-linked trisaccharide is replaced by L-rhamnose, retained robust activity when formulated in DOPC/cholesterol liposomes. Compared under matched formulation conditions, QS-21-Rha induced antibody responses broadly comparable to those elicited by canonical bark-derived QS-21 and stronger responses for several antigen-specific CD4+ T-cell readouts [37]. Together, these results suggest that the effects of glycan substitution depend on the combined influence of monosaccharide identity, linkage configuration, local flexibility, and compatibility with the remaining molecular domains.

### 2.3. Acyl Side Chains: An Adjustable Window Between Immune Stimulation, Membrane Perturbation, and Toxicity

The glycosylated acyl chain of native QS-21 plays a critical role in regulating cellular immunity. Deacylation of *Quillaja* saponins abolished antigen-specific lymphoproliferative activity and shifted the antibody-isotype profile toward a predominantly Th2-type response. Coupling dodecylamine with the carboxyl group of the glucuronic acid residue generated GPI-0100, a hydrolytically stable dodecylamide derivative that induced a Th1-biased antibody profile characterized by increased IgG2a and IgG2b responses, as well as antigen-specific CTL production, while improving hydrolytic stability in aqueous solution and reducing toxicity [16]. Adams et al. showed that amide-stabilized QS analogues bearing simplified glycosylated or non-glycosylated aliphatic chains retained adjuvant activity comparable to that of native QS-21 [38]. Collectively, these findings indicate that an appropriately positioned hydrophobic side chain is an important determinant of saponin adjuvant activity.

Subsequent synthetic studies have refined this understanding into a scaffold-dependent window of side-chain length and geometry. In QS-21-derived analogues, terminal carboxylation or glycosylation improved aqueous solubility while retaining IgG1 and IgG2a responses, whereas introducing an internal polar group weakened IgG2a induction [39]. C14–C16 side chains maintained QS-21-comparable antibody responses in one simplified scaffold [40], whereas C12–C14 chains were more active in analogues retaining the C3-linked disaccharide, although the C14 analogue showed greater toxicity at higher doses [28]. In the *Momordica*-derived VSA-2 scaffold, a simple linear C12 chain was insufficient, while appropriate terminal aromatic functionalization supported humoral and cellular responses [41]. Therefore, side-chain length, polarity, and terminal structure jointly regulate adjuvant activity and safety, and their optimal combination depends on the aglycone and glycan scaffold.

### 2.4. Other Plant-Derived Saponins

Other plant saponins provide independent systems to test whether the structure–activity relationship of QS-21 can be extrapolated.

Comparative studies of structurally related *Platycodon grandiflorum* saponins showed that increasing the number of glucose residues or altering their linkage at C3, as well as removing the terminal apiose residue at C28, reduced in vitro hemolytic activity. However, these changes were generally accompanied by weaker or narrower antigen-specific antibody and cellular immune responses. By contrast, modification of the C24 substituent on the aglycone altered the IgG-subclass profile without reducing hemolytic activity. These findings indicate that the number, linkage, and position of sugar residues, together with the aglycone structure, jointly determine the balance between hemolytic activity and adjuvant activity, and that increasing the number of sugar residues does not necessarily improve adjuvanticity [42].

The lablaboside F analogue S5, which lacks the entire C3-linked trisaccharide while retaining the C28-linked trisaccharide, enhanced OVA-specific total IgG responses in mice. This finding suggests that the C3-linked glycan is dispensable for antibody-enhancing adjuvant activity in this scaffold under the tested conditions [43].

After the formation of VSA-1 and VSA-2 by the introduction of hydrophobic side chains, the activity of *Momordica* saponins is mainly controlled by the side-chain length, terminal geometry and C23 carbonyl group [29,41,44].

Tubeimosides can change cytokines, antigen uptake and lymph node transport at the injection site, but there are multiple differences among natural molecules at the same time, and no single group can be designated as the determinant of a specific immune signal [45].

Collectively, studies of other plant saponins support the limited, rather than universal, transferability of QS-21-derived structure–activity relationships. Glycan architecture, aglycone substitution, hydrophobic side-chain features, and the C23 carbonyl group can differentially influence hemolysis and humoral or cellular immune responses. Additional sugar residues do not necessarily increase adjuvant potency, and some glycan branches may be dispensable. However, these effects are scaffold-dependent, and the concurrent structural differences among natural saponins often preclude assignment of a single structural motif to a specific immune outcome.

### 2.5. Domain Synergy and Hemolysis

The triterpene aglycone core, sugar chain, and side chain are highly coupled in intact saponins. Saponins with relatively close hydrophilic-lipophilic balance (HLB) and similar total IgG levels can still produce different IgG subtype distributions [46]. A single stereochemical change in central connectivity is also sufficient to alter activity [33]. Thus, structural changes first alter conformational distribution, membrane partitioning, and intracellular localization, and subsequently affect innate signalling and antigen presentation, ultimately manifesting as differential antibody and T-cell responses. Amphiphilicity is one of the necessary physical bases, but it is not sufficient to independently predict the type of immune response.

The hemolytic activity of saponins is jointly influenced by their glycan chains, aglycones, and acyl substituents. Sun et al. compared four saponins from *Panax notoginseng* that share the same 20(S)-protopanaxadiol aglycone and observed the following order of hemolytic activity: notoginsenoside K > notoginsenoside R4 > ginsenoside Rb1 > ginsenoside Rd. Their structure–activity analysis indicated that the length of the C20 glycan chain and the glycosylation pattern at C3 influenced hemolytic activity [47]. Savarino et al. found that naturally occurring bidesmosidic saponins from *Chenopodium quinoa* showed no detectable hemolytic activity even at 500 μg/mL, whereas cleavage of the C28 ester-linked glycan converted them into monodesmosidic derivatives with markedly increased hemolytic activity. These findings suggest that C28 glycosylation and the chemical state of the carboxyl group exposed after deglycosylation are important determinants of membrane-disrupting activity [48]. Consistently, alkaline hydrolysis of horse chestnut escins removed the aglycone-bound acyl groups and abolished detectable hemolytic activity, whereas transesterification-associated migration of the acetyl group from C22 to C28 and subsequently to C16 reduced hemolytic activity. These observations suggest that both the degree and position of acylation may modulate saponin-induced membrane disruption, probably by altering molecular amphiphilicity [49].

Collectively, saponin adjuvant activity and hemolytic toxicity are jointly determined by the aglycone, glycan, and acyl domains. A similar hydrophilic-lipophilic balance does not necessarily produce comparable immune profiles, and even a single change in linkage stereochemistry can alter adjuvant activity. Hemolysis is likewise sensitive to glycan number and attachment as well as the degree and position of acylation. Thus, neither amphiphilicity nor any individual structural feature alone is sufficient to predict the immunological activity or membrane-disrupting potential of a saponin.

## 3. Plant-Derived Polysaccharides

Plant-derived polysaccharides have shown considerable potential as vaccine adjuvants, with a growing number of well-characterized preparations shown to enhance antigen-specific immune responses in animal models. However, most vaccine studies have evaluated only a single polysaccharide preparation, and direct in vivo comparisons linking defined structural features to antigen-specific adjuvant performance remain limited. By contrast, more structure–activity studies have investigated the immunomodulatory activity of plant polysaccharides in receptor assays and non-antigen-specific immune models. A critical consideration in these in vitro studies is endotoxin contamination. Residual endotoxin can be quantified using Limulus amebocyte lysate (LAL) or recombinant Factor C assays, while polymyxin B treatment or endotoxin removal columns/filters can help minimize its interference. However, endotoxin testing and control measures remain insufficiently reported in studies of plant polysaccharides. Although such in vitro evidence does not directly establish vaccine adjuvanticity, it provides mechanistic support for their potential as adjuvants. Current studies have mainly associated polysaccharide-induced activation of antigen-presenting cells (APCs) with TLR4-, TLR2-, and Dectin-1-associated pathways.

Among these receptors, TLR4 is the most frequently implicated. In RAW264.7 macrophages, APS upregulated TLR4, MyD88, TRAF6, NF-κB, and AP-1 expression. Pharmacological inhibition and separate TLR4- and MyD88-deficient mouse models supported the involvement of TLR4-mediated, MyD88-dependent signaling [50]. The influence of LPS contamination was excluded using a chromogenic endpoint tachypleus amebocyte lysate assay. Rhamnogalacturonan II from *Panax ginseng* promoted DC maturation and cytokine production in wild-type and TLR2-knockout bone-marrow-derived DCs, but these responses were attenuated in TLR4-knockout DCs [51]. Its ability to induce IL-12 was retained in the presence of polymyxin B, supporting that the observed activity was not primarily attributable to LPS contamination. Similarly, *Rehmannia glutinosa* polysaccharides promoted splenic DC maturation, antigen presentation, and antigen-specific T-cell immunity in wild-type mice, whereas these responses were diminished in TLR4-knockout mice [52]. In this study, the polysaccharide and OVA solutions were passed through an endotoxin-removal column and subsequently filtered through an endotoxin-removal filter before use. Collectively, these findings support a TLR4-dependent component in the immunomodulatory activity of several plant polysaccharides, although they do not establish direct polysaccharide-TLR4 binding.

TLR2 may provide an additional recognition pathway. In RAW264.7 macrophages, blockade of TLR2 reduced nitric oxide production induced by red ginseng acidic polysaccharide (RGAP), while inhibition of ERK or JNK suppressed the same response. RGAP also induced NF-κB and AP-1 activation, supporting the involvement of TLR2-associated ERK/JNK signaling in macrophage activation [53]. Polymyxin B did not inhibit RGAP-induced nitric oxide production, indicating that the observed macrophage activation was unlikely to be primarily caused by LPS contamination.

**Figure 2:**
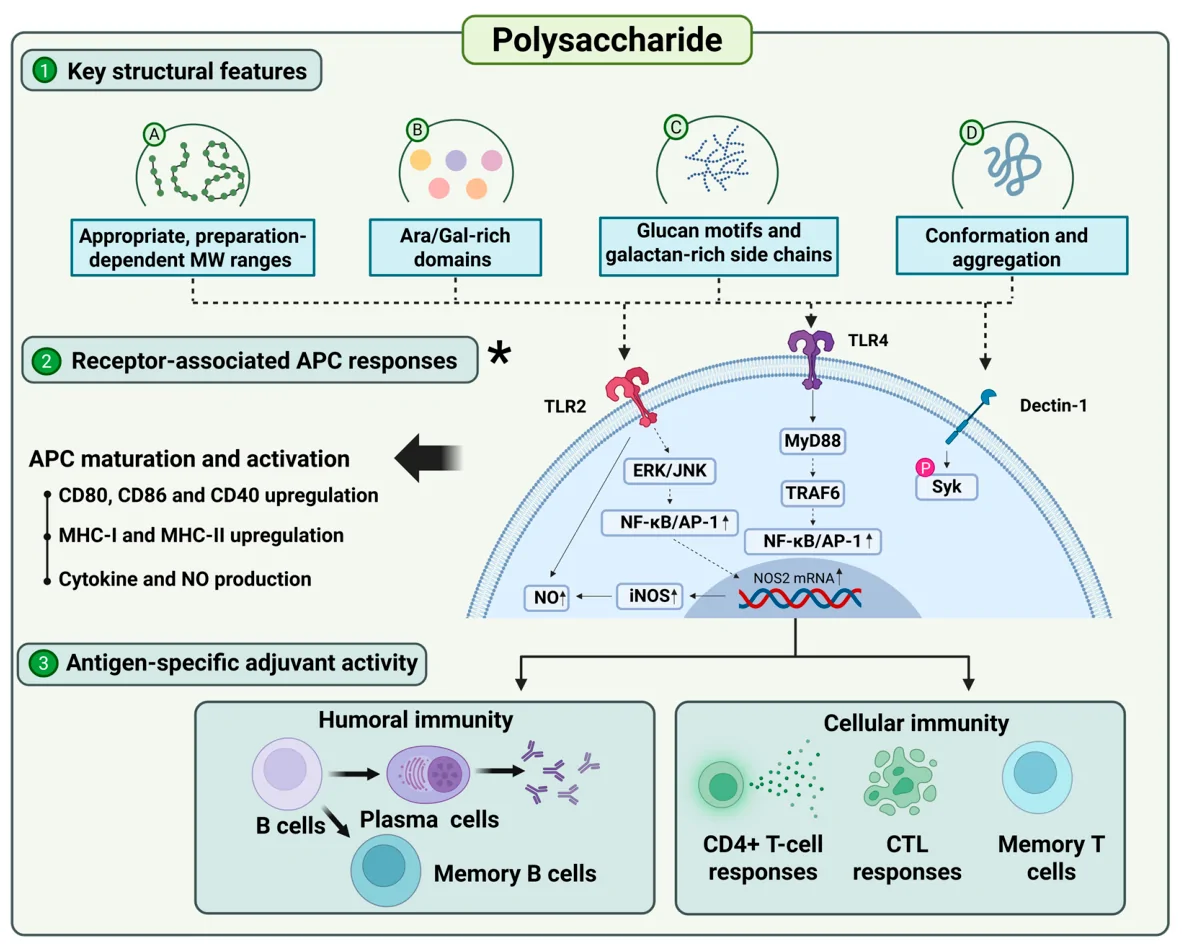
Structural features, receptor-associated APC responses, and antigen-specific adjuvant activity of plant-derived polysaccharides. * Solid arrows indicate experimentally supported pathway order or functional dependence based on receptor blockade, pathway inhibition, or genetic deficiency. Dashed arrows indicate reported associations rather than established causal relationships. The symbol ↑ denotes increased expression or production of the indicated molecules. Plant polysaccharides may participate in multiple PRR-associated pathways. Different polysaccharides may engage different receptors. These events are mechanistic prerequisites, but they do not alone confirm antigen-specific adjuvanticity. AP-1, activator protein 1; APC, antigen-presenting cell; Ara, arabinose; CD, cluster of differentiation; CTL, cytotoxic T lymphocyte; Dectin-1, dendritic cell-associated C-type lectin-1; ERK, extracellular signal-regulated kinase; Gal, galactose; iNOS, inducible nitric oxide synthase; JNK, c-Jun N-terminal kinase; MHC, major histocompatibility complex; MW, molecular weight; MyD88, myeloid differentiation primary response 88; NF-κB, nuclear factor kappa B; NO, nitric oxide; NOS2, nitric oxide synthase 2; PRR, pattern-recognition receptor; Syk, spleen tyrosine kinase; TLR, Toll-like receptor; TRAF6, tumor necrosis factor receptor-associated factor 6.

C-type lectin receptors may also contribute to polysaccharide recognition. In bone-marrow-derived DCs and a dendritic-cell line, *Ficus carica* polysaccharides (FCPS) induced Syk phosphorylation and increased the expression of CD40, CD80, CD86, and MHC-II [54]. Dectin-1 blockade reduced Syk phosphorylation and partially reversed the upregulation of these maturation markers, indicating that FCPS-induced DC maturation is mediated in part through Dectin-1/Syk signaling. In this study, LPS contamination was removed, and the remaining endotoxin level was measured as 0.06 EU/mL using the gel-clot method. Comparative receptor-reporter assays further associated TLR4, TLR2/6, and Dectin-1a activity with several rhamnogalacturonan I (RG-I)-containing pectins bearing neutral galactan or type II arabinogalactan side chains [55]. These preparations were evaluated using both polymyxin B treatment and a recombinant Factor C assay. Polymyxin B did not reduce their receptor-reporter activity, suggesting that LPS was not the primary driver, although the authors noted that contributions from residual LPS could not be completely excluded for some preparations. Together, these observations suggest that structurally heterogeneous plant polysaccharides may depend on overlapping TLR- and C-type lectin receptor-associated pathways rather than a single conserved recognition mechanism. The relative contribution of each pathway appears to vary with polysaccharide structure, preparation, and experimental context. Accordingly, this section first reviews antigen-specific evidence linking polysaccharide structure to vaccine-adjuvant activity and then discusses evidence linking native structural features to immunomodulatory activity and associated recognition pathways (Figure 2).

### 3.1. Structural Features Associated with Antigen-Specific Adjuvant Activity of Plant Polysaccharides

A growing number of preclinical studies have shown that plant-derived polysaccharides can exert substantial vaccine-adjuvant effects. For example, GSPA-0.3, a purified and structurally characterized polysaccharide fraction from *Panax ginseng*, enhanced neutralizing antibody responses to an H1N1 vaccine and promoted DC maturation [56]. Structurally heterogeneous *Astragalus* polysaccharide preparations enhanced antigen-specific responses in influenza and recombinant SARS-CoV-2 vaccine models [57]. In the influenza model, APS-adjuvanted vaccination reduced weight loss and achieved 100% survival following lethal H1N1 challenge, compared with 60% survival in the vaccine-only and alum-adjuvanted groups [57]. Similarly, LCAP, a purified and structurally characterized 27.4 kDa acidic heteropolysaccharide from *Lysimachia christinae*, enhanced antigen-specific humoral and cellular responses to rabies glycoprotein mRNA, influenza, and respiratory syncytial virus vaccines [58,59]. Collectively, these findings support the potential of plant-derived polysaccharides as vaccine adjuvants and warrant further attention to their development. However, studies directly linking defined polysaccharide structures to the activity underlying antigen-specific adjuvant effects remain limited.

Ma et al. compared three chemically characterized water-extractable arabinoxylan fractions prepared from wheat flour, designated WEAX1, WEAX2, and WEAX3, with approximate molecular weights of 87.58, 32.96, and 6.35 kDa, respectively. Their OVA-adjuvant activities were more closely associated with ferulic acid content than with molecular weight [60]. However, because molecular weight, arabinose (Ara) substitution, branching, and ferulic acid content varied concurrently, ferulic acid can only be considered an activity-associated feature rather than an independently validated determinant.

A more direct physical structure–activity relationship has been reported for chicory inulin. Cooper and Petrovsky compared inulin isoforms with the same β-D-(2→1)-polyfructan composition but different supramolecular packing and thermal solubility. The α-inulin isoform, which was soluble at 37 °C, did not significantly enhance hepatitis B surface antigen (HBsAg)-specific antibody responses, whereas the semicrystalline particulate γ- and δ-inulin isoforms exhibited antigen-specific adjuvant activity [61]. These findings associate ordered particulate packing, rather than primary glycan composition alone, with inulin adjuvanticity. Hayashi et al. subsequently showed that delta-inulin particles accumulated in MARCO+ macrophages in draining lymph nodes and that depletion of phagocytic macrophages markedly reduced their antibody-enhancing effect. Delta-inulin also induced DC activation in vivo, and its adjuvant activity was attenuated in TNF-α-deficient mice [62]. Together, these studies connect the semicrystalline particulate state of delta-inulin with macrophage-dependent uptake, indirect DC activation, and TNF-α-associated enhancement of antigen-specific immunity.

### 3.2. Structural Variables Associated with Immunomodulatory Activity

Studies of polysaccharide immunomodulatory activity provide mechanistic support for their potential as vaccine adjuvants and reveal structure–activity relationships. The following subsections discuss how molecular weight, monosaccharide composition, branching and side-chain architecture, and higher-order conformation or aggregation influence APC activation, cytokine production, lymphocyte proliferation, and receptor-associated signals.

#### 3.2.1. Molecular Weight

Molecular weight (MW) may influence polysaccharide immunomodulatory activity by altering physicochemical properties such as solubility and viscosity, thereby affecting cellular uptake, bioavailability, and the activation of APCs and splenic lymphocytes. Comparisons within individual polysaccharide preparations suggest that a relatively active MW range may exist under a given structural background. However, the relationship between MW and activity should be interpreted together with other co-varying structural features. Li et al. found that, among three Astragali Radix polysaccharide fractions of >2000, approximately 10.3, and 0.272 kDa, respectively, the approximately 10 kDa APS-II fraction produced the strongest macrophage, lymphocyte, and immune-organ responses [63]. Jiang et al. similarly found that intermediate-molecular-weight, arabinose-containing *Astragalus* fractions promoted splenic lymphocyte proliferation more strongly than the other fractions examined [64].

In *Lycium barbarum*, Feng et al. found that the >10 kDa fraction induced stronger RAW264.7 macrophage responses than the <10 kDa fraction and underwent clathrin-mediated uptake with lysosomal accumulation [65]. Guo et al. further reported that 100–300 kDa *Lycium barbarum* polysaccharide (LBP) fractions showed stronger TLR4/MD-2 binding and cytokine-inducing activity than fractions below 10 kDa [66]. Together, these studies associate greater immunomodulatory activity with arabinogalactan-rich domains. However, because domain composition, monosaccharide ratios, and branching varied concurrently, the contribution of any single monosaccharide or structural feature cannot be determined independently.

#### 3.2.2. Monosaccharide Composition

Monosaccharide composition and the relative proportions of constituent sugars are closely associated with polysaccharide immunomodulatory activity. Current evidence indicates that higher proportions of arabinose and galactose (Gal) are associated with stronger PRR recognition, particularly TLR4 binding or activation, which may promote macrophage activation, cytokine production, and lymphocyte proliferation. However, because endotoxin contamination can also activate TLR4 and induce similar immune responses, these associations should be interpreted only after appropriate endotoxin testing and controls. Cai et al. found that enzymatically generated low-molecular-weight *Astragalus* fractions retaining α-(1→6)-linked galactose- or arabinogalacto-oligosaccharide branches showed stronger macrophage activation and detectable TLR4 binding, whereas fractions lacking these regions were less active and showed no detectable TLR4 binding [67]. Wang et al. compared two *Astragalus* arabinogalactans of similar molecular weight and found that the highly branched, AG-II-rich APS2 preparation bound TLR4 more strongly and induced greater RAW264.7 cytokine responses than APS1, which contained AG-I and abundant α-glucan structures [68].

Zhang et al. found that partial acid hydrolysis of the *Panax ginseng* RG-I pectin WGPA-2-RG completely removed Ara, partially removed Gal, and reduced its molecular weight. These changes were accompanied by decreased lymphocyte proliferation and macrophage NO production [69]. These results associate Ara/Gal-rich neutral side-chain domains with selected immunomodulatory responses, although the contribution of monosaccharide composition cannot be separated from the concurrent reduction in molecular weight. A broader comparison by Hoel et al. associated RG-I-containing polysaccharides bearing neutral galactan or type II arabinogalactan side chains with activation of human TLR4, TLR2/6, and, for several preparations, Dectin-1a reporter systems, whereas the tested HG-rich polysaccharides were inactive or less active [55]. This cross-preparation comparison supports a domain-level association but remains multivariable.

#### 3.2.3. Branching and Side-Chain Architecture

Branching and side-chain architecture are important structural factors governing polysaccharide immunomodulatory activity. Current evidence indicates that the retention of specific branched motifs and side-chain structures is associated with stronger activity, whereas their selective enzymatic removal can substantially weaken immune responses. These structural features may influence the presentation and recognition of polysaccharides by PRRs, thereby affecting macrophage activation and downstream cytokine production. Li et al. used endo-α-1,4-glucanase to generate APS-II fragments differing in degree of polymerization and retention of branched glucosyl structures. The P4 fraction, characterized by a degree of polymerization (DP) of 10–18 and the highest abundance of branched glucosyl linkages, showed the strongest immunomodulatory activity [70]. These findings associate retained branched glucan structures with activity, but do not isolate branching from concurrent differences in chain length and composition.

More direct evidence comes from the targeted removal of neutral side chains. Grønhaug et al. found that enzymatic removal of β-D-(1→4)-galactan-containing side chains from *Biophytum petersianum* RG-I pectins reduced their activity in Peyer’s patch cells and macrophages [71]. Similarly, Zou et al. showed that endo-1,4-β-galactanase treatment of the *Codonopsis pilosula* RG-I polysaccharide WCP-I reduced IL-6 production by Peyer’s patch cells, whereas removal of terminal arabinose residues had little effect [72]. These targeted enzymatic comparisons support a contribution of galactan-rich side-chain architecture to selected immunomodulatory responses.

#### 3.2.4. Higher-Order Conformation and Aggregation

Higher-order conformation and aggregation can influence polysaccharide immunomodulatory activity by altering solubility, particulate behavior, molecular accessibility, and multivalent presentation to innate immune components. Cooper et al. characterized a series of inulin isoforms with different noncovalent packing and thermal solubility. Among preparations with comparable particle sizes, epsilon and omega inulins activated the alternative complement pathway at least as strongly as delta inulin, consistent with an association between supramolecular organization and innate immunostimulatory activity. However, the isoforms also differed in average degree of polymerization, preventing the observed activity differences from being attributed exclusively to their packing structure [73]. Mikkelsen et al. compared 23 β-glucan preparations, primarily derived from barley and oat, and found that their modulation of IL-12 and IL-10 production in microbially stimulated murine DCs was associated with polymer solubility and aggregation, together with the DP3:DP4 block ratio [74]. Thus, current evidence associates higher-order organization with the immunomodulatory activity of plant polysaccharides, but does not establish conformation or aggregation as an independent causal determinant.

Overall, the most defensible structure-immunomodulation relationships involve preparation-dependent molecular weight ranges, arabinogalactan- or galactan-rich domains, retained glucan sequences and branching, and higher-order organization. However, most evidence derives from multivariable fraction comparisons or non-antigen-specific models. Direct studies that alter one native structural feature, demonstrate receptor engagement, and confirm antigen-specific immunity within the same experimental series remain rare. The structural features of these plant polysaccharides and their relationships with immunomodulatory activity are summarized in Table 1.

## 4. Immune-Enhancement Strategies

Structural modification, delivery engineering and combination strategies enable rational optimization of plant-derived adjuvants tailored to the unique properties of diverse vaccine antigens. Structural derivatization modulates adjuvant receptor recognition, membrane interactions, solubility and stability. Delivery systems control antigen-adjuvant co-localization, lymphatic transport and intracellular fate to coordinate antigen presentation [75,76]. Combination strategies integrate complementary innate immune signals to shape customized immune polarization and support antigen dose sparing in vaccine formulations [75,77].

**Figure 3:**
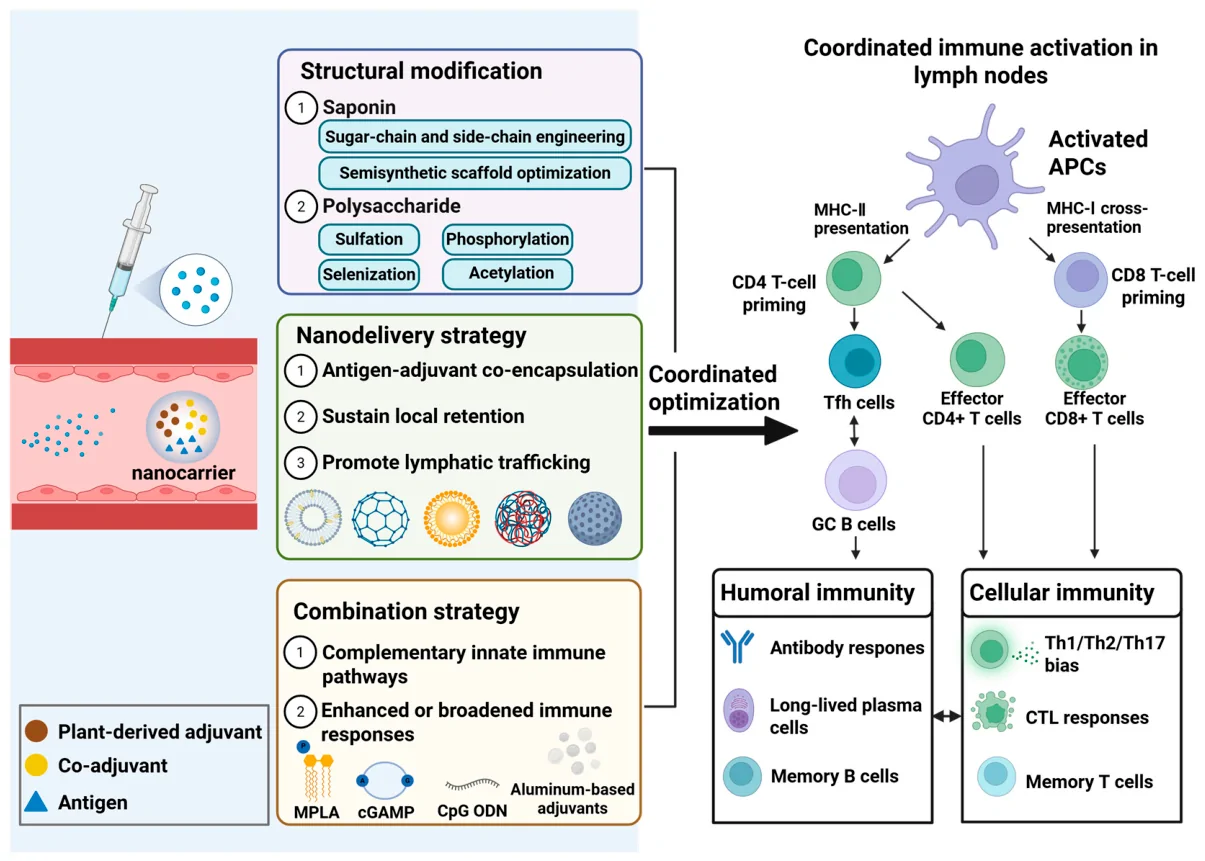
Immune-enhancement strategies for plant-derived saponin and polysaccharide adjuvants. APCs, antigen-presenting cells; CD4, cluster of differentiation 4; CD8, cluster of differentiation 8; cGAMP, cyclic guanosine monophosphate–adenosine monophosphate; CpG ODN, cytosine–phosphate–guanine oligodeoxynucleotide; CTL, cytotoxic T lymphocyte; GC, germinal center; MHC-I, major histocompatibility complex class I; MHC-II, major histocompatibility complex class II; MPLA, monophosphoryl lipid A; Tfh cells, follicular helper T cells; Th1, T helper 1; Th2, T helper 2; Th17, T helper 17.

When paired with vaccine antigens, native saponins and polysaccharides benefit from this modular design: derivatization reshapes their intrinsic physicochemical and immunological characteristics, nanocarriers facilitate co-delivery of antigen and adjuvant, and rational combinations potentiate and broaden vaccine-induced immune responses (Figure 3). Collectively, these strategies offer promising paths to improve immunogenicity, durability of immune responses, delivery performance and safety of plant-derived adjuvants for vaccine applications.

### 4.1. Structural Modification and Derivatization

#### 4.1.1. Structural Modification and Derivatization of Saponins

At the core of saponin modification is the regulation of the balance between hydrophilic sugar chains, triterpene aglycone core, and hydrophobic acyl side chains. In the case of *Quillaja* saponins, N-alkylamide derivatization of the 3-glucuronic acid (GlcA) residue in deacylated *Quillaja* saponins may alter molecular conformation and micellar organization by changing the HLB and associative properties of the molecules [78]. Another route would be to delete nonessential sugar chains, replace readily hydrolyzable ester bonds with stable amide bonds, and simplify the acyl skeleton. MUC1 glycopeptide-T cell epitope three-component conjugates based on this minimal saponin platform can self-assemble into stable aggregates and induce specific IgG that recognize MUC1 on the surface of tumor cells in mice, whereas unconjugated or two-component formulations have weak activity [79].

Semisynthetic derivatization of alternative saponin scaffolds represents another route to QS-21 alternatives. The VSA series obtained by amide side-chain modification of *Momordica cochinchinensis*-derived saponins have been advanced from a model antigen to the SARS-CoV-2 RBD system. VSA-2 induced similar antibodies, Th1-biased responses and protection against challenge as QS-21 in mice, suggesting that the source of the parent nucleus can be replaced, but the spatial configuration of key sugar chains and hydrophobic side chains must be preserved [80,81]. Engineered yeast has been able to synthesize QS-21 and its structural analogues, providing conditions for systematically changing the steps of glycosylation and acylation on the same production platform [82].

#### 4.1.2. Structural Modification and Derivatization of Polysaccharides

Polysaccharide derivatization mainly affects conformation, PRR multivalent recognition and cellular uptake by introducing charged groups, changing hydrophilicity, or regulating chain length and branching. Sulfation is currently one of the most common structural modification strategies for plant polysaccharides. Wusiman et al. isolated a homogeneous *Lagenaria siceraria* polysaccharide fraction, LSP50, with a reported carbohydrate content of 98.6%, and subsequently prepared its sulfated derivative, sLSP50_-1.5_, with a degree of substitution (DS) of 1.5. The derivative showed enhanced NDV-specific humoral and cellular immune responses in a Newcastle disease virus vaccine model, which could maintain high hemagglutination inhibition antibody titers and NDV-specific IgG levels. In addition, sLSP50_-1.5_ also induced the expression of Th1-type cytokines such as IFN-γ and TNF-α and Th2-type cytokines such as IL-4 and IL-6, suggesting that sulfation enhances the ability of LSP50 to induce mixed humoral and cellular immune responses [83]. The sulfate group may promote receptor or membrane protein interactions by increasing the negative charge and changing the chain conformation.

Selenization combines the effect of trace elements with the polysaccharide skeleton and can increase the immunostimulatory potential of polysaccharides. Qiao et al. extracted Chinese Angelica polysaccharide (CAP) from *Angelica sinensis* by hot-water extraction and ethanol precipitation, followed by deproteinization, dialysis, and chromatographic purification, obtaining a purified but polydisperse polysaccharide preparation rather than a single chemically defined molecular entity. Compared with unmodified CAP, selenylated Chinese Angelica polysaccharide (sCAP) promoted DC maturation and antigen presentation and enhanced MyD88/NF-κB signaling [84]. Likewise, Wang et al. obtained heterogeneous *Brassica rapa* polysaccharide preparations by ultrasonic-assisted aqueous extraction, trichloroacetic-acid deproteinization, dialysis, and freeze-drying. Selenylated *Brassica rapa* polysaccharides containing Se-O-C linkages enhanced humoral, cellular, and intestinal mucosal immune responses to a live-attenuated Newcastle disease vaccine in chickens relative to unmodified polysaccharides [85]. Acetylation regulates immune recognition by altering hydroxyl exposure, hydrophobicity, and conformation. Zhang et al. used CPP_0.1_, a polysaccharide fraction obtained from *Cyclocarya paliurus* leaves by aqueous extraction and DEAE-cellulose chromatography with 0.1 M NaCl elution, and prepared its acetylated derivative, Ac-CPP_0.1_. Ac-CPP_0.1_ was reported to be a high-purity, homogeneous polysaccharide mainly composed of arabinose and galactose. Acetylated *Cyclocarya paliurus* polysaccharide Ac-CPP_0.1_ promoted DC maturation, as indicated by increased CD80, CD86, and MHC-II expression and cytokine secretion, and alleviated cyclophosphamide-induced immunosuppression in mice. These effects were associated with activation of the TLR4/NF-κB pathway [86].

Phosphorylation introduces negatively charged, hydrophilic groups into polysaccharide chains, thereby improving water solubility and altering their physicochemical properties and conformation [87]. Liu et al. found that phosphorylation of *Atractylodes lancea* polysaccharide ALP-1 increased its solubility and thermal stability, reduced crystallinity, and altered its molecular dimensions and morphology. The resulting derivative, P-ALP-1, showed the strongest capacity to stimulate NO production in RAW264.7 macrophages, indicating that phosphate-group introduction enhanced its macrophage-stimulatory activity [88].

### 4.2. Delivery System

Licensed human vaccines containing plant-derived saponin adjuvants use formulated adjuvant systems rather than unformulated free saponins. AS01 incorporates QS-21 and MPL into cholesterol-containing liposomes and is used in Shingrix, Mosquirix and Arexvy, whereas Matrix-M, which comprises nanoparticles formed from separately purified *Quillaja saponaria* saponin fractions, cholesterol and phospholipids, is used in Nuvaxovid. These particulate formulations reduce direct exposure of cell membranes to free saponins and improve formulation stability. Plant-derived polysaccharide translation remains limited by structural heterogeneity, incomplete compositional definition and batch-to-batch variability.

Beyond the licensed liposomal and matrix-type formulations described above, delivery platforms investigated for plant-derived adjuvants include immune-stimulating complexes (ISCOMs), oil-in-water emulsions, polymeric nanoparticles, and inorganic nanocarriers based on calcium or silica materials [11]. Depending on the platform and formulation design, antigens and adjuvants may be encapsulated, adsorbed, surface-functionalized, or combined with preformed particles. These approaches can improve physicochemical stability and regulate release, tissue distribution, lymphatic transport, and cellular uptake.

#### 4.2.1. Formulation and Nanodelivery of Saponin Adjuvants

Free unformulated QS-21, when simply mixed with antigen, exhibits well-defined dose-limiting toxicities in preclinical models. In vitro, QS-21 induces approximately 50% hemolysis of sheep erythrocytes at 7–9 μg/mL, stemming from cholesterol-dependent pore formation in cell membranes [82]. In mice immunized with GD3–KLH, synthetic SQS-21 preparations caused dose-dependent body-weight loss. At 50 μg, mice receiving the individual SQS-21-Api or SQS-21-Xyl isomers also showed ruffled fur and hunched posture, and one death occurred in each isomer group [89]. Free QS-21 has additionally been associated with local pain, inflammation and tissue injury, whereas incorporation of QS-21 into liposomes or ISCOM-like particles reduces its hemolytic activity and local reactogenicity while preserving its immunostimulatory properties [82,90]. Licensed AS01-containing vaccines have shown acceptable safety profiles, with reported adverse reactions being predominantly transient local and systemic reactogenicity [91]. A previous study incorporated *Quillaja brasiliensis* saponin fraction QB-80 into ISCOM-like nanoparticles (IMXQB-80), and the complete control setting confirmed that nanoencapsulation significantly reduced hemolysis and cytotoxicity toward L-929 cells compared with free QB-80, while retaining potent antigen-specific immune responses even at a quarter of the conventional saponin dosage [92].

Self-nanoemulsifying delivery systems also represent a powerful strategy to address the safety concerns associated with free plant saponins. Luo et al. developed an ophiopogonin D (OPD)-loaded self-nanoemulsifying adjuvant system, which effectively reduced the membrane-mediated cytotoxicity of free OPD against dendritic cells by encapsulating hydrophobic saponins within nanoemulsion cores. This formulation prolonged antigen retention at the injection site and elicited comprehensive Th1, Th2 and Th17 immune responses, achieving superior prophylactic and therapeutic anti-tumor effects compared with free saponin and blank nanoemulsion groups [93]. Such formulation design isolates extracellular free saponins to attenuate cytotoxicity, while retaining intracellular immunostimulatory functions.

Nanoparticle formulations can also coordinate the delivery of QS-21 with complementary immunostimulants. Luan et al. co-encapsulated QS-21 and CpG ODNs in ionizable lipid nanoparticles for a varicella zoster virus glycoprotein E (gE) subunit vaccine. Compared with the corresponding unencapsulated CpG/QS-21 combination, the nanoparticle formulation increased the serum gE-specific IgG endpoint titer by approximately 2.1-fold and increased the frequencies of gE-responsive, Th1-cytokine-producing CD4+ T cells by approximately 3.5–3.7-fold [94]. However, the study did not include a group receiving gE with empty LNPs. Therefore, the contribution of the LNP components themselves cannot be completely separated from the effects of delivering CpG ODNs and QS-21 in the same particles. Silva et al. developed a saponin/MPLA nanoparticle adjuvant (SMNP) that increased lymphatic flow and promoted antigen entry into draining lymph nodes. SMNP also enhanced antigen uptake by cognate B cells and supported GC B-cell, Tfh-cell, and antibody responses [95]. These studies indicate that particulate co-formulation can improve both the safety of QS-21 and the coordinated delivery of mechanistically complementary adjuvants.

#### 4.2.2. Formulation and Nanodelivery of Polysaccharide Adjuvants

Nanoformulation can coordinate the delivery of polysaccharide adjuvants and antigens to draining lymph nodes and antigen-presenting cells. Li et al. extracted two Astragali Radix polysaccharide fractions with molecular weights of approximately 12.19 kDa and 135.67 kDa. Astragali Radix polysaccharide fractions and OVA_257-264_ peptide were prepared by microfluidic control and coated with a sheddable calcium phosphate layer to form self-adjuvanting nanocomposites, in which polysaccharides simultaneously performed antigen assembly and immune stimulation functions, and enhanced DC uptake, antigen-specific CD8+ T-cell responses and tumor control [96]. Shen et al. used a commercially obtained *Polygonatum sibiricum* polysaccharide (PSP) preparation and generated microparticulate PSP (MP-PSP) through physical adsorption onto calcium carbonate particles. Microparticulation of PSP with calcium carbonate delayed antigen release, promoted TLR4-related DC activation and Tfh-B-cell interaction, and showed a stronger adjuvant effect than free polysaccharide [97].

Polymeric nanoparticles can enhance the delivery and adjuvant activity of polysaccharides. The Chinese yam polysaccharide (CYP) used by Luo et al. was a single polysaccharide component obtained from Chinese yam by hot-water extraction, Sevag deproteinization, high-speed counter-current chromatography, and Sephadex G-100 size-exclusion chromatography. PLGA nanoparticles co-encapsulating CYP and OVA effectively promoted DC activation in draining lymph nodes and induced stronger humoral and cellular immune responses than those observed in the free CYP combination group and blank PLGA carrier group, directly confirming the independent adjuvant enhancement of polysaccharides rather than carrier-derived effects [98]. Similarly, Wusiman et al. obtained a homogeneous neutral polysaccharide fraction (AHP) from Alhagi honey polysaccharides. AHP encapsulated by PLGA exhibited a superior capacity to promote lymphocyte proliferation and Th-type cytokine secretion compared with free polysaccharides and blank nanoparticle controls, further validating the reliable adjuvant activity of nanodelivered polysaccharides [99].

Recent formulations also combine polysaccharide adjuvants with inorganic nanocarriers to improve antigen transport and cellular uptake. He et al. used a purified acidic polysaccharide from *Cistanche deserticola*, designated CDP, which the authors characterized as a homogeneous polysaccharide with a weight-average molecular weight of approximately 132.1 kDa. *Cistanche deserticola* polysaccharide-functionalized dendritic fibrous nanosilica nanoparticles (CDP-DFNS) enhanced transepithelial antigen transport, dendritic-cell uptake and activation, and BSA-specific systemic and mucosal immune responses following oral immunization, while blank DFNS carriers failed to produce equivalent immune enhancement, confirming that the mucosal and systemic immune improvement was specifically derived from polysaccharide-mediated immune regulation rather than carrier material activity [100]. Gu et al. obtained total Chinese yam polysaccharides by hot-water extraction and ethanol precipitation and developed Chinese yam polysaccharide-loaded aluminum oxyhydroxide nanoparticles (CYP-Al NPs). Compared with free CYP, including a tenfold higher dose, and commercial Alhydrogel, the complete CYP-Al NP formulation promoted dendritic-cell and germinal-center B-cell activation and induced durable OVA-specific antibody responses, including IgG2a, together with enhanced CTL-associated responses [101]. However, because the in vivo study did not include OVA plus unloaded Al NPs, these findings support the performance of the complete CYP-Al NP formulation but do not isolate the contribution of the aluminum oxyhydroxide carrier.

In summary, nanoformulation and carrier delivery enable plant-derived saponin and polysaccharide adjuvants to elicit stronger and more persistent antigen-specific immune responses compared with free adjuvants. These optimized effects stem from multiple synergistic mechanisms: protecting adjuvants and antigens from hydrolytic and biological degradation, sequestering membrane-lytic saponins to reduce cytotoxicity, achieving sustained antigen release, prolonging local antigen retention, and realizing spatial co-localization of adjuvants and antigens. Additionally, precise particle size regulation and surface functionalization facilitate lymphatic trafficking, mucosal barrier penetration, antigen-presenting cell uptake and intracellular antigen processing.

### 4.3. Combination Strategy

Plant-derived natural ingredients often have multitarget immunomodulatory effects, and combining them with other immune adjuvants can further increase the complexity of the immunostimulatory signal [102]. Different adjuvant components can simultaneously act on PRRs, intracellular innate immune sensing, antigen uptake and presentation, and immune cell interaction in lymph nodes, thus forming an interconnected immune activation network. The concurrent engagement of these signals may amplify early innate immune stimulation and enhance the activation of APCs and costimulatory signals. Thus, combination strategies may produce stronger immune-enhancing effects than individual adjuvants in some experimental settings. In particular, with the in-depth understanding of the mechanism of action of adjuvants, an increasing number of combination systems have begun to utilize complementary immunostimulatory mechanisms to broaden vaccine-induced immune responses.

#### 4.3.1. Strategy of Saponins Combined with Adjuvants

The overall goal of saponin-based combination strategies is to leverage complementary innate immune signals to increase the strength and quality of antigen-specific responses and, as appropriate, to improve immune bias, enhance immune potency, or reduce the required dose. AS01 co-formulates QS-21 with the TLR4 agonist MPL in liposomes, thereby amplifying the early IFN-γ-related innate response and further enhancing antibody and Th1-type immunity, effects that cannot be fully reproduced by either component alone [24]. Shaik et al. combined the synthetic TLR4 agonist FP20 with the simplified synthetic QS-21 variant QS21v, forming a co-formulation with a mean particle size of approximately 110–120 nm. In an OVA model, the combination containing 10 μg of each component induced higher antigen-specific IgG responses than either component administered alone at the same dose [103]. Luna et al. incorporated QS-21 and the fully synthetic TLR4 agonist E6020 into cholesterol-containing liposomes to generate SPA14. In mice and cynomolgus macaques immunized with an hCMV vaccine candidate containing glycoprotein B and the pentameric complex, SPA14 induced durable virus-neutralizing antibodies, antigen-specific memory B cells, and IFN-γ- and IL-4-secreting T-cell responses. These responses were generally comparable to those elicited by AS01B [104]. In parallel, non-clinical studies showed that SPA14 did not cause systemic toxicity in repeated administration experiments in rabbits. Separate safety-pharmacology studies found no adverse effects of intramuscular SPA14 on respiratory function in beagle dogs or of subcutaneous E6020 on cardiovascular function in cynomolgus macaques [105].

The combination of saponin adjuvant and STING agonist may be a strategy to improve the vaccine efficacy in older or immunosenescent populations. Vassilieva et al. showed that, in aged mice immunized with an H1N1 haemagglutinin subunit vaccine, cGAMP alone provided no survival benefit and Quil-A alone conferred only partial protection, whereas their combination achieved complete protection after intramuscular immunization and enhanced vaccine-specific antibody responses [106]. When incorporated with the H1N1 vaccine into dissolving microneedle patches, the same combination also achieved complete survival in 21-month-old mice and reduced maximal post-challenge weight loss from approximately 16% with the unadjuvanted vaccine to 3% [107]. These findings support the complementary activity of saponins and cytosolic STING agonists in aged animals.

#### 4.3.2. Strategy of Polysaccharides Combined with Adjuvants

Combining polysaccharide adjuvants with aluminum-based adjuvants may enhance selected antigen-specific immune responses compared with the corresponding single-component or control formulations. Zhang et al. used a commercially supplied *Astragalus* polysaccharide (APS) preparation and found that adding APS to a SARS-CoV-2 KP.2 recombinant protein vaccine formulated with Rehydragel^®^ aluminum hydroxide gel enhanced antigen-specific antibody responses, increased the frequencies of IgG+ germinal-center B cells and Tfh cells, and promoted antigen-stimulated cytokine production by CD4+ and CD8+ T cells compared with the corresponding formulation containing Rehydragel^®^ alone [108]. Zhao et al. isolated Paeoniae Radix Alba polysaccharides (PRAPs) as a relatively homogeneous, low-polydispersity α-glucan with a reported molecular weight of 14.391 kDa. PRAPs activated macrophages primarily through TLR2/TLR4-mediated, MyD88-dependent NF-κB signaling. In an OVA immunization model, combining PRAPs with alum induced higher antigen-specific antibody levels and a stronger Th1 response than alum alone [109]. These findings suggest that plant polysaccharides capable of activating PRR-associated signaling may serve as functional complements to aluminum-based adjuvants by preserving strong humoral immunity while broadening cellular responses and supporting immunological memory. Overall, combination adjuvants should be rationally selected according to the immune responses required for protection.

Beyond aluminum-based adjuvants, polysaccharide adjuvants can also be combined with defined pattern-recognition receptor agonists to broaden the antigen-specific immune profile. Honda-Okubo et al. compared the delta-inulin adjuvant Advax alone with Advax combined with CpG55.2, a Toll-like receptor 9 (TLR9) agonist, in a recombinant H7 hemagglutinin vaccine model. Advax predominantly induced H7HA-specific IgG1, whereas Advax-CpG55.2 additionally induced IgG2a, IgG2b, and IgG3. Thus, compared with Advax alone, the combination broadened the antigen-specific antibody-isotype profile and promoted a more Th1-associated immune response [110]. The translational feasibility of this combination was subsequently supported by SpikoGen, a recombinant SARS-CoV-2 spike vaccine formulated with Advax-CpG55.2, which demonstrated efficacy and acceptable tolerability in a randomized Phase III trial [111].

## 5. Challenges and Future Prospects

### 5.1. Challenges in the Development of Plant-Derived Vaccine Adjuvants

The primary barrier to the translation of plant-derived adjuvants is not a shortage of candidates, but the absence of a verifiable quality-control framework linking structural heterogeneity and batch-to-batch consistency to biological potency. Species, geographical origin, harvest season, plant part, extraction and purification procedures, and storage conditions can alter key structural characteristics of saponins and polysaccharides [82,112]. For complex natural mixtures, it is difficult to ensure that the key quality attributes are consistent with immune function only by using total sugars, total saponins or fingerprint similarity indicators. Quality control should gradually shift to structural monomers or defined component ratios, and high-resolution mass spectrometry, nuclear magnetic resonance, chromatography, multi-angle light scattering, and advanced conformational analysis should be combined with cell-based potency assays to establish structure–activity traceability relationships.

The second challenge concerns the safety margin of plant-derived adjuvants. Hemolysis and membrane perturbation of saponins, endotoxin or protein residues, and excessive inflammation in combination with PRR agonists may misrepresent general immune stimulation as a beneficial adjuvant effect. Candidates should be evaluated separately for local reactogenicity, systemic cytokines, hemocompatibility, toxicity of repeated administration, reproductive risks, and risks in special populations. These evaluations should also distinguish transient innate activation from persistent tissue damage. Especially for mucosal routes such as the nasal cavity, it is necessary to pay attention to the cross-barrier distribution of the adjuvant and the risk of neurotoxicity [113].

The third issue is the translation from animal models to humans. PRR expression, cellular composition, dose scale, and immune senescence differ among species, and antibody elevations in model antigens are not equivalent to target disease protection. In the future, more human whole-blood, PBMC, organoid or lymphoid tissue models should be added, and systemic immunological indicators should be used as soon as possible to connect animals with early clinical responses.

### 5.2. Computationally Assisted Structure–Activity Analysis and Derivative Design

A clearer understanding of structure–activity relationships may enable computational methods to guide the derivatization of plant-derived adjuvants. Molecular docking and related approaches can help prioritize structural modifications and identify candidate motifs that may contribute to receptor-associated activity. This strategy has been applied to the screening of inulin derivatives, followed by experimental evaluation of the selected derivative in vitro and in vivo [114]. For structurally defined saponins, computationally guided modification may similarly support the design of semisynthetic or synthetic analogues with optimized adjuvant activity.

The application of these methods to polysaccharides nevertheless requires particular caution. Their structural heterogeneity, conformational flexibility, uncertain three-dimensional organization and multivalent interactions cannot be adequately represented by conventional docking to a single static structure. Future studies should therefore combine computational screening with selective enzymatic hydrolysis, controlled debranching or chemoenzymatic reconstruction of polysaccharides obtained from the same source and production batch, while keeping other structural variables as consistent as possible. Molecular dynamics simulations and conformational ensemble approaches may then be used to prioritize the smallest glycan motifs most likely to retain activity. These predictions require subsequent validation by direct binding assays, such as surface plasmon resonance or isothermal titration calorimetry, together with receptor-deficient cells, receptor-site mutations and relevant in vitro and in vivo models. Such an integrated strategy could provide more specific targets for polysaccharide extraction, controlled degradation and derivatization.

For saponins, advances in synthesis provide a complementary route for converting structure–activity information into defined analogues. Tobacco and engineered yeast platforms have enabled the biosynthesis of QS-21 [115,116], while characterization of the soapwort saponin pathway has expanded the available glycosylation and acylation enzymes [117]. Together with semisynthesis and computational analysis, these platforms may facilitate the production and comparative evaluation of structurally defined analogues and help identify modifications associated with improved adjuvant activity.

### 5.3. Regulatory Considerations and Clinical Translation

Regulatory evaluation focuses on the final adjuvanted vaccine rather than on the natural component in isolation. WHO guidelines require nonclinical evaluation of adjuvants and adjuvanted vaccines according to product characteristics and clinical use, paying attention to ingredient interactions, dose, local and systemic toxicity, and relevant animal models [118]. The current clinical evaluation guidelines of EMA further emphasize safety, immunogenicity, protective efficacy or effectiveness, and immune protection-related indicators [119].

Clinical translation of plant-derived saponins is currently more extensive and mature than that of polysaccharides. Non-particulate QS-21 administered with the GM2-KLH vaccine reached Phase III evaluation and induced antigen-specific antibody responses. However, two randomized trials failed to demonstrate clinical benefit [120,121]. These outcomes cannot be attributed to QS-21 alone because vaccine efficacy also depended on the antigen, disease setting, and comparator. Nevertheless, they showed that strong immunogenicity was insufficient for successful translation. Incorporation of QS-21 into liposomal combination systems, particularly AS01, subsequently provided a well-controlled platform for reproducible delivery and complementary immune stimulation, contributing to the approval of several vaccines. Matrix-M further demonstrates that standardized *Quillaja* saponin fractions can be translated successfully through a defined nanoparticle platform. These experiences indicate that potent immunostimulatory activity alone is insufficient for translation; formulation design, compositional control, scalable manufacturing, and antigen-specific clinical validation are equally important. In comparison, most plant-derived polysaccharides remain at the preclinical stage, partly because their structural heterogeneity complicates characterization, potency testing, and batch consistency. Delta inulin represents an important exception. Its development as a reproducible microcrystalline particulate adjuvant, Advax, together with its favorable tolerability and compatibility with different antigens, has supported Phase I/II evaluation. Its use with CpG55.2 in an emergency-authorized vaccine in Iran further illustrates the potential of a standardized polysaccharide platform. The translational development of polysaccharide-based adjuvants is not limited to plant-derived polysaccharides, as fungal polysaccharides have also been extensively investigated. MABH-9002b, comprising the shiitake mushroom-derived β-glucan lentinan complexed with a synthetic CpG ODN, has been evaluated as an adjuvant for the RSV vaccine candidate VN-0200 in Phase I and II clinical trials [122]. Table 2 compares currently approved adjuvant systems and candidates that have completed clinical evaluation.

Translational strategies should consider manufacturability simultaneously at the candidate discovery stage. Complex natural mixtures require stable supply chains, raw material identification, impurity limits and stability. Multi-component nanoformulations also need to be controlled for particle size distribution, entrapment efficiency, free adjuvant, release, and freeze–thaw stability. Structural simplification, semisynthesis, and synthetic biology may improve supply reliability, but process-related structural differences and impurities require careful characterization [80,116]. Establishing scalable processes and stabilizing key quality attributes early generally reduces translational risk more than redesigning formulations in late preclinical stages.

## 6. Conclusions

Overall, plant-derived saponins and polysaccharides are promising vaccine-adjuvant candidates, but the current understanding of their structure–activity relationships differs between the two classes. Structure–activity studies of plant-derived saponins have focused predominantly on QS-21 and its analogues, showing that the aglycone, sugar chains and side chain jointly regulate adjuvant activity and toxicity. For polysaccharides, current studies mainly associate structural features with immunomodulatory responses, supporting their adjuvant potential while leaving direct links to antigen-specific adjuvanticity to be further established. Most available evidence remains preclinical, particularly for heterogeneous plant polysaccharide preparations, and causal structure–activity relationships and direct receptor-binding evidence remain limited. Structural modification, delivery systems, and combination strategies may improve stability, antigen-adjuvant co-encapsulation, and immune responses. Future development should prioritize structurally defined preparations, consistent manufacturing, controlled comparisons, and mechanistic and protective validation. These approaches may facilitate the development of safe plant-derived adjuvants with predictable immune functions. However, such progress will depend on rigorous quality, safety, and clinical evaluation.

## Figures and Tables

**Table 1:** Representative plant-derived polysaccharides and structure-immunomodulatory activity relationships.

Plant Source	Fraction	MW (kDa)	Monosaccharide Composition	Experimental Model	Structure-Immunomodulatory Activity Relationship	Ref.
Astragali Radix; dried root	APS-I	>2000	Rha:GalA:Gal:Glc:Ara = 0.10:0.39:13.4:17.2:1	Murine splenic lymphocytes; murine peritoneal macrophages; YAC-1 target cells; cyclophosphamide-immunosuppressed mice, with subsequent ex vivo assessment of immune cells	APS-II showed the strongest overall enhancement of lymphocyte proliferation, LPS-induced total IgG production, macrophage phagocytosis, and NK-cell cytotoxicity. APS-II improved hematological parameters, spleen and thymus indices, and delayed-type hypersensitivity responses, and restored lymphocyte proliferation, cytokine production, antibody-forming responses, macrophage phagocytosis, and NK-cell activity. APS-I and APS-III generally showed weaker effects.	[63]
	APS-II	10.3	Rha:GalA:Gal:Glc:Ara = 0.14:0.14:9.6:24.04:1			
	APS-III	0.272	Rha:GalA:Gal:Glc:Ara = 0.375:0.375:18.8:90.5:1			
Astragali Radix; root water extract	APS1	257.7	Glc only	Spleen lymphocytes	APS2 and APS3 enhanced splenocyte proliferation, whereas APS1 and APS4 showed no significant stimulatory effects.	[64]
	APS2	40.1	Ara only			
	APS3	15.3	Rha:Glc:Gal:Ara = 1:10.76:6.55:12			
	APS4	3.2	Gal:Ara = 3.02:1			
*Lycium barbarum* fruit	LBP < 10 kDa	6.99	Glc 47.27%, Ara 0.81%, Gal 1.20%	RAW264.7 macrophages	The >10 kDa fraction increased cell viability-assay signals, whereas the <10-kDa fraction showed little activity. The >10-kDa fraction altered macrophage morphology and polarization markers, increased NO, TNF-α, IL-6, and ROS production in the absence of LPS, but reduced their excessive production under LPS stimulation.	[65]
	LBP > 10 kDa	27.71	Glc 11.68%, Ara 27.76%, Gal 13.37%			
*Lycium barbarum* fruit	Seven ultrafiltration fractions of LBP	Fractions spanning <10 to >1000	Ara-, Gal- and Glc-containing heteropolysaccharides; proportions changed with size fractionation/digestion	THP-1 cells; SPR assays	In PMA-differentiated THP-1 macrophage-like cells, 100–300 kDa LBP was associated with greater cytokine production than <10 kDa fractions. In SPR assays, >10-kDa fractions were associated with stronger TLR4/MD-2 binding.	[66]
*Astragalus membranaceus* root	APS-A1	0.609	Gal:Ara:Glc = 1:1.84:78.36	RAW264.7 macrophages; BLI assays	In RAW264.7 macrophages, branch-retaining APS-A1 and APS-G3 increased NO production and TNF-α and IL-6 mRNA expression, whereas APS-G1 and APS-G2 showed no significant effects on NO production. In biolayer interferometry assays, APS-A1 and APS-G3 were associated with detectable TLR4 binding, whereas APS-G1/G2 showed little binding.	[67]
	APS-G1	0.651	Glc only			
	APS-G2	1.315	Glc only			
	APS-G3	4.487	Man:Ara:Gal:Glc = 1:2.66:4.45:10.99			
*Astragalus membranaceus* roots from two geographical origins	APS1	106.5	Man:Rha:GalA:Glc:Gal:Ara = 0.79:1.70:3.28:79.07:6.63:8.56	RAW264.7 macrophages; SPR assays	In RAW264.7 macrophages, AG-II-rich APS2 showed greater phagocytosis and IL-6, IL-1β and TNF-α mRNA expression than APS1. In SPR assays, APS2 was associated with higher TLR4 affinity.	[68]
	APS2	114.5	Man:Rha:GalA:Glc:Gal:Ara = 1.61:6.05:10.21:6.34:27.39:48.39			
*Panax ginseng* root	WGPA-2-RG	Approximately 110	Ara:Gal:Glc:Rha:GalA:GlcA = 40.9:44.4:2.9:4.1:5.3:2.0	Murine splenic lymphocytes; murine peritoneal macrophages	In splenic lymphocytes, WGPA-2-RG enhanced splenocyte proliferation, whereas its degradation product RG-6H-I showed no stimulatory effect. In peritoneal macrophages, RG-6H-I was associated with reduced NO induction while retaining phagocytosis-stimulating activity.	[69]
	RG-6H-I	Approximately 40	Ara:Gal:Glc:Rha:GalA:GlcA = 0:76.1:0.6:10.1:8.6:4.6			
Multiple plant tissues	GP-AP	300.5	Preparation-dependent; included GalA-rich, Ara/Gal-rich and Xyl/Ara-rich compositions	HEK-Blue cells; THP1-Blue cells; bone marrow-derived macrophages	In HEK-Blue cells, RG-I-containing GP-AP and DMP-ED-I activated hTLR2/6, hTLR4, and hDectin-1a, whereas A-A2-I and FRAGP showed receptor-selective activation. HG-rich preparations showed limited or no activation, and arabinoxylan-rich LCP was inactive toward the tested PRRs. GP-AP, DMP-ED-I, and FRAGP also enhanced NF-κB reporter activity in THP1-Blue cells and promoted NO production in mouse bone marrow-derived macrophages with IFN-γ costimulation.	[55]
	A-A2-I	67				
	DMP-ED-I	40.3				
	FW	34.5				
	CAP	392				
	CPA	70				
	FRAGP	<9.3				
	LCP	190				
*Astragali Radix* root	P1–P4	P4, 1.639–2.935; MWs of P1–P3 not reported	P1–P3 were >98% Glc; P4 contained 85.67% Glc with minor GlcA, Rha, GalA, Gal and Ara	RAW264.7 macrophages; murine splenic lymphocytes; YAC-1 target cells	P4 showed the strongest overall enhancement of macrophage phagocytosis, NK-cell cytotoxicity against YAC-1 cells, conA-induced lymphocyte proliferation, and total IgG secretion. P4 also enhanced LPS-induced lymphocyte proliferation, whereas P1–P3 showed no apparent effects on this response.	[70]
*Biophytum petersianum* aerial parts	BPII, BPII.1, BPII.2 and their enzyme-treated derivatives	NR	BPII: predominantly GalA; BPII.1 and BPII.2: predominantly GalA, Rha and Gal, with Ara, Xyl, Glc, GlcA and minor sugars	Peyer’s patch cells; peritoneal macrophages	In Peyer’s patch cells and peritoneal macrophages, β-(1→4)-galactan degradation in BPII.1/BPII.2 was associated with reduced IL-6 production. In these models, degradation of β-(1→3,6)-galactan reduced macrophage IL-6 responses to both fractions, but reduced Peyer’s patch-cell responses only to BPII.2.	[71]
*Codonopsis pilosula var. modesta* root	WCP-I	Not numerically reported	Ara:Rha:Man:Gal:Glc:GalA = 5.5:6.4:0.7:17.6:0.2:69.6	In vitro: Peyer’s patch cells In vivo: mice	In vitro: in ConA-stimulated Peyer’s patch cells, WCP-Ia and WCP-Ib showed stronger IL-6 stimulation than WCP-I. WCP-Ib, obtained by removing terminal Ara residues from WCP-Ia, retained IL-6-stimulatory activity. WCP-Ic, obtained by subsequent β-(1→4)-galactan degradation, showed reduced activity. In vivo: WCP-Ia increased the spleen index, serum IL-6, TNF-α, and TGF-β levels, and the CD4^+^/CD8^+^ T-cell ratio in Peyer’s patches. WCP-I increased intestinal sIgA levels, whereas WCP-Ia showed no significant effect on this endpoint.	[72]
	WCP-Ia		Ara:Rha:Man:Gal:Glc:GalA = 13.0:10.6:0.8:37.5:0.3:37.8			
	WCP-Ib		Ara:Rha:Man:Gal:Glc:GalA = 10.1:11.1:0.5:42.3:0.6:35.4			
	WCP-Ic		Ara:Rha:Man:Gal:Glc:GalA = 11.0:12.6:0.6:38.1:0.7:37.0			
Commercial chicory inulin (Raftiline HP)	Amorphous inulin; AI-1; AI-2; GI; DI; ZI; EI; OI	Not directly reported in kDa	Predominantly Fru, with terminal Glc	Cell-free human serum complement assay with rabbit erythrocyte lysis	In a cell-free human serum complement assay, EI and OI showed complement-activating activity at least as strong as that of DI.	[73]
Barley and oat grains; four non-cereal reference β-glucans	Nineteen cereal mixed-linkage β-glucans; curdlan, cellulose, lichenan, and yeast β-glucan	130–410	Glc (β-glucan homopolymers)	Bone marrow-derived DCs	In LPS-stimulated bone marrow-derived dendritic cells, lower solubility and a higher DP3/DP4 ratio were associated with greater IL-10 responses.	[74]

In Ref. [55], endotoxin testing and a polymyxin B (PMB) control were reported. In Ref. [74], low endotoxin contamination was inferred from cytokine responses rather than established by direct endotoxin quantification. Endotoxin testing or control measures were not reported in the other studies included in this table. AG-II, type II arabinogalactan; Ara, arabinose; BLI, biolayer interferometry; CD4, cluster of differentiation 4; CD8, cluster of differentiation 8; ConA, concanavalin A; DCs, dendritic cells; DP3/DP4, ratio of trisaccharide to tetrasaccharide fragments released by lichenase digestion; Fru, fructose; Gal, galactose; GalA, galacturonic acid; Glc, glucose; GlcA, glucuronic acid; hDectin-1a, human dendritic cell-associated C-type lectin-1a; HEK-Blue cells, human embryonic kidney 293-derived reporter cells; HG, homogalacturonan; hTLR, human Toll-like receptor; IFN-γ, interferon gamma; IgG, immunoglobulin G; IL, interleukin; kDa, kilodalton; LPS, lipopolysaccharide; Man, mannose; MD-2, myeloid differentiation factor 2; MW, molecular weight; NF-κB, nuclear factor kappa B; NK, natural killer; NO, nitric oxide; NR, not reported; PMA, phorbol 12-myristate 13-acetate; PMB, polymyxin B; PRRs, pattern-recognition receptors; Ref., reference; RG-I, rhamnogalacturonan I; Rha, rhamnose; ROS, reactive oxygen species; sIgA, secretory immunoglobulin A; SPR, surface plasmon resonance; TGF-β, transforming growth factor beta; THP-1 cells, a human monocytic leukemia cell line; THP1-Blue cells, THP-1-derived NF-κB reporter cells; TLR4, Toll-like receptor 4; TNF-α, tumor necrosis factor alpha; Xyl, xylose; YAC-1 cells, a murine lymphoma cell line used as a target for NK-cell cytotoxicity assays.

**Table 2:** Regulatory and clinical development status of vaccine adjuvant systems and candidates containing plant-derived saponins and polysaccharides.

Adjuvant System or Candidate	Formulation and Adjuvant Composition	Development Stage	Vaccine, Antigen and Representative Regimen	Ref.
AS01B	Liposomal adjuvant system containing QS-21 and MPL	Component of a licensed vaccine (Shingrix)	Shingrix: recombinant VZV glycoprotein E, 50 µg + AS01B containing 50 µg QS-21 and 50 µg MPL	[123]
AS01E	Liposomal adjuvant system containing QS-21 and MPL	Component of licensed vaccines (Arexvy and RTS,S/AS01E)	Arexvy: RSVPreF3, 120 µg + AS01E containing 25 µg QS-21 and 25 µg MPL; RTS,S/AS01E: RTS,S antigen, 25 µg + AS01E containing 25 µg QS-21 and 25 µg MPL	[124,125]
Matrix-M	Nanoparticle adjuvant system containing *Quillaja saponaria* Fraction A and Fraction C (85:15) with cholesterol and phosphatidylcholine	Component of licensed vaccines (Nuvaxovid and R21/Matrix-M)	Nuvaxovid: recombinant SARS-CoV-2 spike protein, 5 µg + Matrix-M, 50 µg; R21/Matrix-M: R21 malaria antigen, 5 µg + Matrix-M1, 50 µg	[126,127]
ISCOMATRIX	Antigen-free saponin–lipid nanoparticle system comprising purified *Quillaja saponaria* saponins, cholesterol and phospholipid	Phase II	Recombinant NY-ESO-1, 100 µg + ISCOMATRIX, 120 µg	[128]
AS02A	Oil-in-water emulsion adjuvant system containing QS-21 and MPL	Phase I/II	Recombinant Mtb72F, 40 µg + AS02A containing approximately 50 µg QS-21 and 50 µg MPL	[129]
AS15	Liposomal multicomponent adjuvant system containing QS-21, MPL, and CpG 7909	Phase III	Recombinant MAGE-A3, 300 µg + AS15 containing 50 µg QS-21, 50 µg MPL, and 420 µg CpG 7909	[130]
ALFQ	Liposomal adjuvant system containing DMPC, DMPG, cholesterol, synthetic MPLA (3D-PHAD) and QS-21	Phase I	SARS-CoV-2 spike ferritin nanoparticle antigen, 25 or 50 µg + ALFQ containing 200 µg 3D-PHAD and 100 µg QS-21	[131]
GLA-LSQ	Liposomal adjuvant system containing GLA and QS-21	Phase I	Recombinant full-length *Plasmodium falciparum* circumsporozoite protein, 10, 30, or 60 µg + GLA-LSQ containing 5 µg GLA and 2 µg QS-21	[132]
GPI-0100	Semisynthetic mixture of glucuronic-acid-modified derivatives of deacylated *Quillaja* saponins	Phase I/Ib	EC90 vaccine (KLH–FITC conjugate), 1.2 mg + GPI-0100, 3.0 mg	[133]
Advax	Particulate polysaccharide adjuvant consisting of microcrystalline delta inulin	Phase I/II	Jack Jumper ant venom, 25 or 50 µg + Advax, 5 mg	[134]
Advax-CpG55.2/SpikoGen^®^	Combination adjuvant system containing Advax and CpG55.2	Market-authorized for emergency use in Iran following Phase III evaluation	Recombinant SARS-CoV-2 spike protein, 25 µg + Advax, 15.5 mg + CpG55.2, 171 µg	[111]

Abbreviations: 3D-PHAD, 3-deacyl phosphorylated hexaacyl disaccharide; ALFQ, Army Liposome Formulation containing QS-21; CpG, cytosine-phosphate-guanine; DMPC, 1,2-dimyristoyl-sn-glycero-3-phosphocholine; DMPG, 1,2-dimyristoyl-sn-glycero-3-phospho-(1′-rac-glycerol); FITC, fluorescein isothiocyanate; GLA, glucopyranosyl lipid A; ISCOM, immune-stimulating complex; KLH, keyhole limpet hemocyanin; MAGE-A3, melanoma-associated antigen 3; MPL, 3-O-desacyl-4′-monophosphoryl lipid A; MPLA, monophosphoryl lipid A; Mtb72F, *Mycobacterium tuberculosis* 72F fusion protein; NY-ESO-1, New York esophageal squamous cell carcinoma 1; RSVPreF3, respiratory syncytial virus prefusion F protein antigen; SARS-CoV-2, severe acute respiratory syndrome coronavirus 2; VZV, varicella-zoster virus.

## Data Availability

No new data were created or analyzed in this study. Data sharing is not applicable to this article.
